# Supramolecular Organogels Based on Cinnarizine as a Potential Gastroretentive System: In Vitro and In Silico Simulations

**DOI:** 10.3390/gels12010058

**Published:** 2026-01-08

**Authors:** Masar Basim Mohsin Mohamed, Ghaidaa Hameed, Mohanad Naji Sahib, Zainab Kadoori, Hasanain Shakir Mahmood, Aqeel Abdulridha Khudhair

**Affiliations:** 1 Department of Pharmaceutics, College of Pharmacy, Mustansiriyah University, Baghdad 10052, Iraq; ghaidaahameed@uomustansiriyah.edu.iq (G.H.); a.a.khudhair@uomustansiriyah.edu.iq (A.A.K.); 2 Department of Pharmaceutics, College of Pharmacy, Al-Farabi University, Baghdad 10022, Iraq; 3 Baghdad Health Directorate-Al Karkh, Ministry of Health and Environment, Baghdad 10090, Iraq; zsaad6254@gmail.com; 4 Department of Pharmaceutics, College of Pharmacy, University of Alkafeel, Najaf 54001, Iraq; hasanain.sh@alkafeel.edu.iq

**Keywords:** cinnarizine, Gastroplus^®^ software, in situ gelation, organogels, self-assembly

## Abstract

(1) Background: Gastroretentive systems are an interesting option for enhancing the bioavailability of weak bases and poorly soluble drugs. The aim of this study was to formulate supramolecular organogels based on cinnarizine (CIN) as a potential gastroretentive system. (2) Methods: The organogels were prepared with different oils in different ratios. Thereafter, their pharmaceutical characteristics and in vitro gastric retention were evaluated through in vitro and in silico simulations. (3) Results: Organogels with different proportions of CIN to oils were successfully obtained. The DSC thermal analysis results demonstrated that all organogels showed gel–sol temperature transitions. The frequency sweep test verified that all organogels presented frequency-independent behavior. Optical imaging revealed longitudinal spherulites of the 1:4 CIN in organogels in all oils. The CIN organogels in all oils (1:4) were observed to float in gastric media during the entire release study. The pharmacokinetic parameters of CIN in peppermint oil (1:4) revealed a close Cmax value to that of the 25 mg immediate-release tablet, but a different AUC. (4) Conclusions: The organogels in all oils floated throughout the release study, establishing their potential as a gastroretentive system. Furthermore, these dosage forms were assessed as a gastric-controlled system through in silico simulations, which enabled prediction of their pharmacokinetic parameters.

## 1. Introduction

An organogel is a viscoelastic semi-solid material that results from the self-assembly of small molecules (gelators) into a supramolecular structure to generate 3D scaffolds via non-covalent interactions [1]. The hydrophilic groups of the gelators in organic solvents promote this self-assembly—mainly through hydrogen bonding—to form organogels. Moreover, organogelation occurs when the non-covalent interactions between gelator molecules are greater than the interactions between the gelator and the solvent [2]. In the pharmaceutical field, organogels are used for different routes of administration, including oral [3,4], topical [5] and many other drug delivery systems [6].

Indeed, many compounds with no medical activity—such as fatty acids and surfactants (sorbitan palmitate and sorbitan mono- or tristearate)—are known to act as small-molecule gelators in addition to steroids and peptides [7,8,9,10,11,12,13]. Interestingly, a few drugs also act as gelators to form organogels, serving simultaneously as a carrier delivery system and a bioactive material and, thereby, reducing the need for excipients. For example, two studies have shown that folic acid gelled an N-methyl pyrrolidone–water mixture and propylene glycol, respectively [4,14]. Additionally, doxorubicin gelled water in the presence of salts [15]. Moreover, previous studies have investigated piperazine derivatives as small-molecule gelators [16,17]. The current study explores the applicability of cinnarizine (CIN, a piperazine derivative) as a small-molecule gelator, in addition to its pharmacological activity [18,19,20,21].

CIN—a weak base and poorly soluble drug—has pH-dependent solubility and variable bioavailability after oral administration. However, previous studies have shown that gastroretentive systems are an interesting option for enhancing its bioavailability [22,23]. Many studies have prepared CIN as part of a gastroretentive system to control its release rate in the upper part of the intestine [22,24,25,26]. However, no study has investigated the use of CIN in organogel formation. Considering the medical importance of CIN and its status as a piperazine derivative, we investigated the use of CIN as a small-molecule gelator for the first time in this study; in particular, to gel peppermint oil (PO), sesame oil (SO), medium-chain triglyceride oil (MCT) and lemon oil (LO). Furthermore, we investigate efficacy of the semi-solid organogel formulas for oral administration, monitor their gastroretentive properties concurrently and assess their pharmacokinetics via an in silico physiologically based pharmacokinetics (PBPK) approach in combination with their in vitro drug release profiles.

Several studies have recently utilized in silico physiologically based pharmacokinetics (PBPK) in combination with in vitro drug release profiles to predict in vivo pharmacokinetic parameters [27,28]. Hence, the second aim of this study was to construct a model of a 25 mg CIN immediate-release tablet for oral delivery using the Gastroplus software, based on the lysosomal trapping concept. This PBPK model was then used to predict the pharmacokinetic parameters of the CIN-prepared organogels from in vitro drug release data as a floating CIN delivery system.

## 2. Results and Discussion

### 2.1. Organogel Formulation

**Figure 1:**
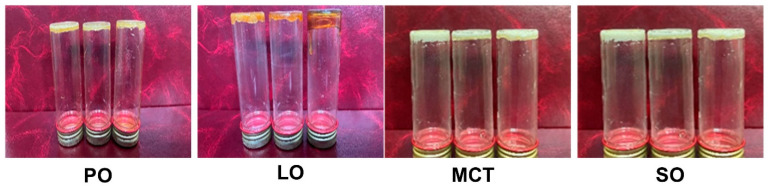
Organogels gelated at room temperature after being heated in a water bath at 90 °C. From left to right in all image, the cinnarizine:oil ratios are 1:4, 1:6 and 1:8 (where PO, LO, MCT and SO denote peppermint oil, lemon oil, medium-chain triglyceride oil and sesame oil, respectively).

Figure 1 shows the successfully obtained organogels. The gelation time after the vials cooled showed a negative relationship with the CIN concentration, as presented in Supplementary File S1. All organogels appeared as semi-solid gels except for the CIN:LO mixture (1:8), which formed a viscous liquid that flowed; as the aim of this study was to formulate a semi-solid organogel that possesses solid-like behavior for oral administration using a capsule as a carrier, this mixture was excluded from further evaluation. In addition, most organogels exhibited syneresis, except those with a 1:4 ratio of CIN:SO and CIN:MCT. This syneresis may be due to incomplete branching of the CIN growing scaffold, which aids in the retention of oils [29].

### 2.2. Thermoreversible Gelation and Organogel In Situ Formation Studies

**Figure 2:**
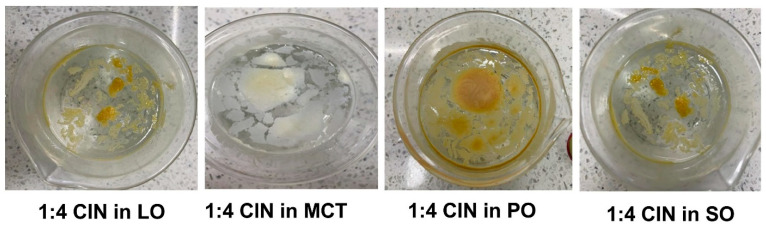
In situ gelation of 1:4 CIN organogels in 200 mL of water after adding the liquid organogels obtained by adding 50 µL of ethanol to 1:4 CIN in LO, MCT, PO or SO. As illustrated, no precipitation occurred and the oils were retained by CIN as a gelator, indicated by the floating organogel masses.

We investigated the thermoreversibility of the organogels, represented by the transfer ability of the CIN organogels from different statuses after thermal impact. All successfully obtained organogels were thermoreversible (from liquid to solid status and vice versa), as they gelled after being subjected to a second melting exposure. Furthermore, the liquid 1:4 CIN organogels with all oils solidified in situ, forming organogel masses that once more retained the oils 20 min after pouring them into 200 mL of water at ambient temperature (Figure 2). Semi-solid CIN organogels formed after ethanol had escaped from the liquid organogel formulas, as the ethanol functioned to disrupt the bonds that form during gelation, rendering the formulations as liquids. The above results reveal that CIN successfully acted as a low-molecular-weight gelator, showing an in situ gelation ability with PO, SO, MCT and LO. These results are consistent with those of a previous study, where N-lauroyl-L-alanine methyl ester gelled soybean oil after liquefication with ethanol [8]. In summary, the CIN organogels were thermally reversible and the CIN molecules were able to self-assemble after ethanol liberation in water, forming a semi-solid mass and retaining the oils in an organogel form without precipitation.

### 2.3. DSC

**Figure 3:**
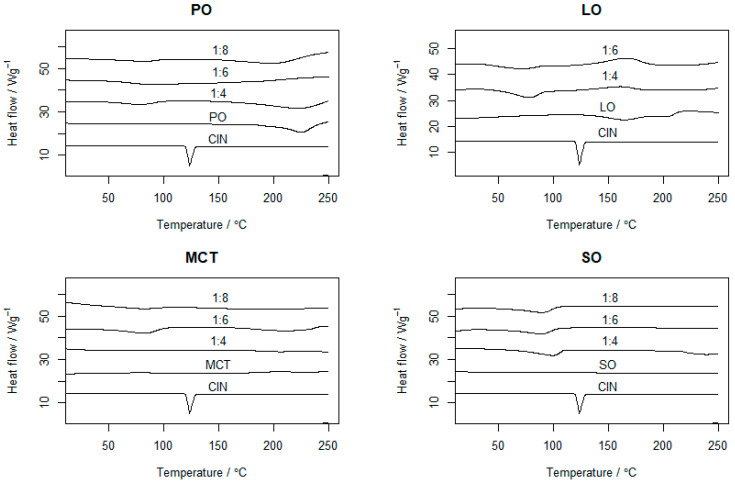
The DSC thermograms of pure CIN, PO, LO, MCT, SO and corresponding organogels in different proportions (1:4, 1:6 and 1:8). Each run ranged from 0 to 300 °C with a heating rate of 10 °C/min.

The thermal behaviors of the CIN organogels in the different oils were evaluated via DSC. Figure 3 shows the thermograms of CIN, oils and the organogels of CIN with different oils. CIN showed a sharp endothermic peak at 123.5 °C, nearly identical to that in a previously published study, indicating its crystallinity and purity [30]. The PO thermogram in Figure 3 indicates oil evaporation between 200 and 300 °C, which is comparable with the previous study [31]. For the CIN:PO organogels, the sharp endothermic peak of CIN disappeared and the PO evaporation peak reduced, with the appearance of gel–sol transition peaks at 78.5, 90 and 80.98 °C for the 1:4, 1:6 and 1:8 organogels, respectively.

On the other hand, the LO thermogram showed a shoulder exothermic peak, indicating a phase transition of the oil and its recrystallisation. In the thermograms of the 1:4 and 1:6 CIN:LO organogels, the gel–sol transition temperatures were 77.6 and 70.34 °C, respectively. Meanwhile, for MCT, the gel–sol transition temperatures for the 1:4, 1:6 and 1:8 organogels appeared at 89.52, 83.51 and 80.98 °C, respectively. Finally, for the 1:4, 1:6 and 1:8 CIN:SO organogels, the gel–sol transition temperatures were 99.11, 90.34 and 89.34 °C, respectively.

All prepared organogels showed gel–sol transition peaks much higher than body temperature (i.e., 37 °C), indicating that they would remain in a semi-solid state while in the stomach media, and thus, reduced the body’s thermal impact on the organogel. Additionally, the gel–sol transition temperature peaks were lower than the melting point of CIN. This observation is similar to that reported for gels based on doxorubicin, which showed lower transition temperatures (ranging from 37.85 to 48.85 °C) at organogel concentrations from 15 to 30 mM in 0.25 M NaCl, when compared with the melting point of pure doxorubicin (229–231 °C) [15].

### 2.4. Oscillatory Rheology Study

#### 2.4.1. Amplitude Sweep

**Figure 4:**
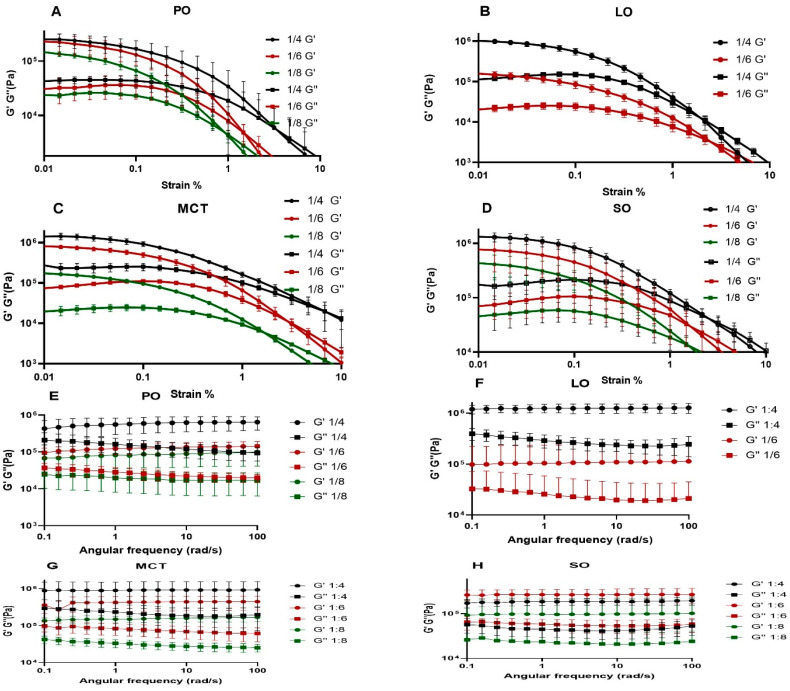
(**A**–**D**) Amplitude sweep figures of CIN organogels; and (**E**–**H**) frequency sweep figures. Each is the average of 3 runs and was performed at a temperature of 37 °C. For the amplitude sweep study, the angular frequency of 10 rad/s and the strain range was from 0% to 100%; the frequency sweep study was carried out within the angular frequency range from 0 to 100 rad/s.

All prepared organogels exhibited high differences (one order of magnitude) between their G′ and G″ values, providing further evidence for organogel formation (Figure 4A–D and Supplementary File S2) [2]; in particular, when G′ >> G″, the organogel type is indicated [32]. Furthermore, the organogels showed increases in G′, G″ and flow point values with an increasing CIN concentration in the formulation. The LVER values were almost identical except for that of CIN:SO (1:4), which showed a slightly increased value in comparison with the other formulations. The G′ values of CIN organogels were similar to those of organogels prepared from 12-hydroxystearic acid with span 40 and 60 in castor oil—a formula which successfully slowed the release of diclofenac sodium in small intestinal media [33]. The G′ values of CIN organogels were similar to those of 5 and 10% *w*/*w* 12-hydroxystearic acid in sesame oil organogels (around 95,683 and 442,796 Pa), which were also formulated as a gastroretentive system [34]. Considering the similarity of our results to those reported in these studies, the CIN organogels were concluded to have gastroretentive potential.

#### 2.4.2. Frequency Sweep

This test was performed to identify the effect of the frequency rate of oscillatory motion on the organogels. As seen in Figure 4E–H, the 1:4, 1:6 and 1:8 CIN organogels in PO, LO, MCT and SO exhibited parallel G′ and G″ curves, which did not intersect at any point, suggesting that the obtained CIN organogels are frequency-independent. These results are similar to those reported for organogels prepared with 5 to 25% *w*/*w* of glyceryl monostearate in MCT and high-oleic sunflower oil [35]. Furthermore, organogels with a formulation of 4% *w*/*w* diosgenin in canola oil, developed for oral use, showed parallel G′ and G″ curves, as well as similar G′ values to those obtained for the 1:8 CIN in PO and 1:6 CIN in LO organogels in this study [36].

The oscillatory studies indicated the suitability of the CIN organogels with different oils for oral delivery, based on their strength (as represented by the G′ values) and the frequency-independent nature.

### 2.5. Fourier-Transform Infrared (FTIR)

FTIR analysis aids in confirming the existence of bonds between molecules that ensure successful organogel formation. As demonstrated in Supplementary File S3, the full-range spectrograms of CIN, oils and the prepared organogels present two peaks at 1134 and 1592 cm^−1^ associated with C-N and C=C stretching [37]. These groups might be responsible for the solubility of CIN in the oils and its capacity for self-assembly, as a balance between these two processes is necessary for the formation of organogels. The CIN organogel in MCT showed a noticeable peak shift from 1134 cm^−1^ to 1151 cm^−1^ and 1158 cm^−1^ in the spectrograms at all ratios (1:4, 1:6 and 1:8), which corresponded to C-N stretching. This shift was not observed for the 1:4, 1:6 and 1:8 CIN:PO, CIN:LO and CIN:SO organogels, as presented in Supplementary File S3.

Due to the higher melting temperatures obtained in the DSC study by the 1:4 CIN:MCT and CIN:SO organogels, as well as their higher G′ values—reflecting both the thermal stability and elasticity or strength, which suggests organogel durability, and thus, potential for oral route intake [38], specifically as a gastroretentive system—all 1:4 CIN organogels were chosen for the next part of this study.

### 2.6. Optical Light Microscopy

**Figure 5:**
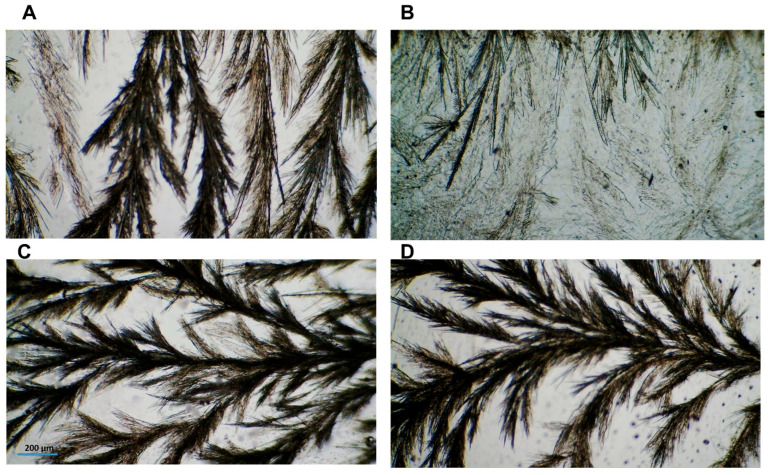
(**A**–**D**) Optical images of 1:4 CIN in PO, LO, MCT and SO, respectively. Images were captured using a 4× objective and scaled against 200 µm.

The ability of CIN molecules to self-assemble and the resultant scaffold morphology were observed. Captured images of the selected organogels (1:4 CIN in all oils) are shown in Figure 5A–D. Interestingly, the CIN organogels showed the same fiber structure in all oils; i.e., the spherulites grew longitudinally, in a structured manner. However, the 1:4 CIN in the LO scaffold was not dense or transparent in appearance. These structures resembled the spherulites of carnauba wax ester in a fatty alcohol organogel [39]. Moreover, the morphology results are consistent with those of previous reports [32,40] regarding organogel formation, which further confirms the oscillatory rheology results mentioned above.

### 2.7. In Vitro Release Study

**Figure 6:**
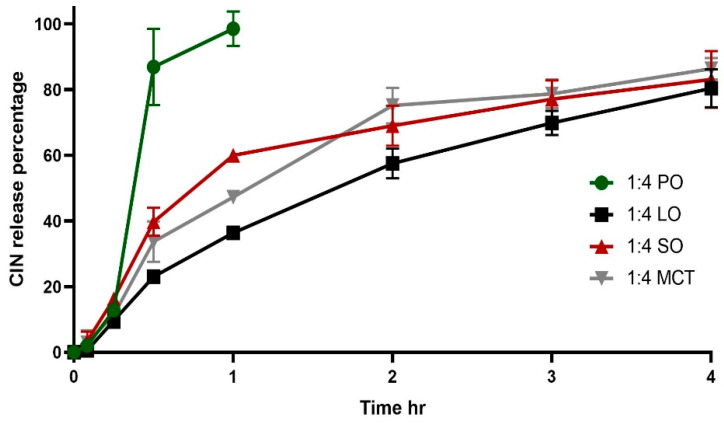
In vitro release of 1:4 Cinnarizine organogels in 900 mL 0.1 N HCL (pH 1.2) at 37 °C. Each profile is an average of three trials ± SD. SO, MCT, LO and PO denote sesame oil, medium chain triglyceride oil, lemon oil and peppermint oil, respectively.

In the release study, the organogels remained present after dissolution of the hard gelatin capsules: the organogels were observed to float throughout the entire release experiment’s duration with gradual disappearance in the acidic media, which is considered a favorable medium for CIN release and solubility [41]. The floating of the organogels can be attributed to their high oil contents, which helped the formulations to remain buoyant in the gastric media. In a previous study, organogels were formulated from beeswax and carnauba wax in various oils (e.g., corn, cottonseed and soybean), and it was reported that the formulated organogels remained suspended and floating in the gastric fluid [42]. As illustrated in Figure 6, 1:4 CIN in PO showed rapid release, whilst the 1:4 CIN in LO, MCT and SO organogels presented comparable release profiles, which were slower than that of the 1:4 CIN:PO organogel. The results from a repeated measures ANOVA demonstrated that the 1:4 CIN:PO organogel had a significantly different release profile compared with the other organogels (*p* < 0.05), while the other formulations did not show any significant differences between their profiles (*p* > 0.05).

The slower release rate of the latter organogels can be correlated to their higher G′ values, which were 1,321,600, 1,410,266 and 1,410,266 Pa for 1:4 CIN in SO, MCT and LO, respectively, compared with the lower G′ of 249,906 Pa for 1:4 CIN in PO. High G′ values reflect robustness, leading to slow release. This has been shown for organogels obtained based on a low content of glycerol monostearate combined with glycerol monopalmitate, where those that presented a higher complex modulus G* were observed to slow the release of 5-FU in gastric media [43].

### 2.8. Physiologically Based Simulation of CIN Pharmacokinetics

**Figure 7:**
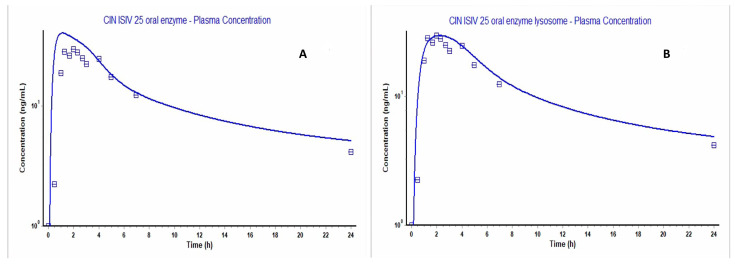
Gastroplus figures, where the dotted and solid lines representing the observed and calculated values, respectively. (**A**) The predicted versus observed plasma concentration–time curve before applying lysosome entrapment; (**B**) that after choosing the PBPKPlus (Lukacova with lysosomes) modules.

The parameters and inputs in Table 1 were applied to construct a model using Gastroplus^®^ (version 9.8.2), as shown in Figure 7A, which did not closely fit the observed clinical plasma concentration–time curve; specifically, around the Tmax and Cmax values. CIN’s physical properties—i.e., log P of more than 5 [44] and being a weak base with pKa of 7.38, according to the ADMET predictor—suggest the occurrence of lysosomal entrapment. Considering these properties, CIN can passively permeate cell and lysosome membranes but subsequently becomes trapped within lysosomes due to protonation of CIN and the low lysosome pH of 4 [45,46]. Hence, the PBPK Plus module was chosen to select the perfusion-limited tissue–Lukacova with lysosomes setting, and the Vss after lysosome entrapment was changed to 833 (L).

For further optimization of the calculated concentration plasma–time curve, the unbound fraction in the enterocyte was optimized by conducting parameter sensitivity analysis to integrate it into the lysosomal model. The reduced value of the unbound fraction in enterocytes was less than 100%, indicating a low rate of drug movement to the portal vein from the enterocytes. This led to a prolonged time to reach the maximum drug concentration in the blood [47]. As shown in Supplementary File S4, the unbound fraction in enterocyte values that matched the observed Tmax and Cmax values ranged from 6.4% to 10% and from 4.7% to 8%, respectively; hence, 8% was selected due to its best fit. In this context, the implementation of lysosomal entrapment better matched the delayed Tmax value, as well as decreasing Cmax to be close to the observed Cmax (Table 2); this could be attributed to the lysosome’s enzyme digestion action, according to Nee Ling et al. [48].

The outcome was a model that fit the observed data on the plasma concentration–time curve well, as shown in Figure 7B, and evidenced by the fold error of the Cmax, Tmax and AUC_0-t_ values. As detailed in Table 2, the pharmacokinetic parameters were within an acceptable value (i.e., not more than 2).

The fit of the constructed model was once more validated with respect to another oral in vivo observation investigating the Stugeron^®^ 25 mg tablet [49], which showed close Cmax, Tmax and AUC_0-t_ values (30.624 ng/mL, 2.32 h, and 289.21 ng-h/mL, respectively) to the calculated ones, as clarified in Table 1 and Supplementary File S4. In a different in vivo study, Stugeron 25 mg showed different Cmax, Tmax and AUC_0-t_ values; namely, 41.34 ng/mL, 3 h and 292.35, respectively [50]. This difference could be justified by the different ethnicities of the study participants, which may have resulted in variations in pharmacokinetic parameters. Furthermore, the enzyme CYP2D6, which highly metabolizes CIN and exhibits a known polymorphism, either leads to over- or under-metabolism, which, in turn, leads to differences in pharmacokinetic parameters between individuals [51,52].

**Table 1:** AFll inputs used to construct the oral CIN model for the 25 mg immediate-release tablet.

Parameter	Value and Unit	Reference of Value
2B6 enzyme input in PBPK	Km 17.2 (µm) Vmax 1.75 (pmol/min/pmol)	[53]
2D6 enzyme input in PBPK and GUT	Km 2.44 (µm) Vmax 0.72 (pmol/min/pmol)	[53]
1A2 enzyme input in PBPK	Km 219.063 (µm) Vmax 6.965 (nmol/min/nmol)	ADMET_ Predictor 10.4
2C19 enzyme input in PBPK and GUT	Km 18.822 (µm) Vmax 13.848 (nmol/min/nmol)	ADMET_ Predictor 10.4
CLsys	3.913 (L/h)	ADMET_ Predictor 10.4
Vss	831.931 (L)	ADMET_ Predictor 10.4
Molecular weight	368.53 (g/mole)	ADMET_ Predictor 10.4
Peff	1 (cm/s × 10^−4^)	[54]
Log P	5.01	ADMET_ Predictor 10.4
Solubility	0.0000855 (mg/mL) at pH = 8.35	ADMET_ Predictor 10.4
Blood/plasma conc. ratio	0.74	ADMET_ Predictor 10.4
Fup %	4.19	ADMET_ Predictor 10.4
Drug particle density	1.2 (gm/mL)	ADMET_ Predictor 10.4
Diffusion coefficient	0.62 (cm^−2^/s × 10^−5^)	ADMET_ Predictor 10.4
Particle size radius	25 (µm)	ADMET_ Predictor 10.4
Adj Plasma Fup %	0.292	ADMET_ Predictor 10.4

**Table 2:** The observed and calculated pharmacokinetic parameters of a 25 mg immediate-release oral tablet with and without application of lysosomal entrapment.

Pharmacokinetics Parameters (Units)	Observed Values	Calculated Values Without Lysosomal Entrapment	Calculated Values with Lysosomal Entrapment	Fold Error Values Without Lysosomal Entrapment	Fold Error Values with Lysosomal Entrapment
Cmax (ng/mL)	29.472	40.05	29.445	1.35	1
Tmax (h)	2.0001	1.12	2.24	1.78	1.11
AUC_0-inf_ (ng-h/mL)	338.08	498.88	451.83	1.47	1.33
AUC_0-t_ (ng-h/mL)	272.89	300.91	274.61	1.1	1.001

**Figure 8:**
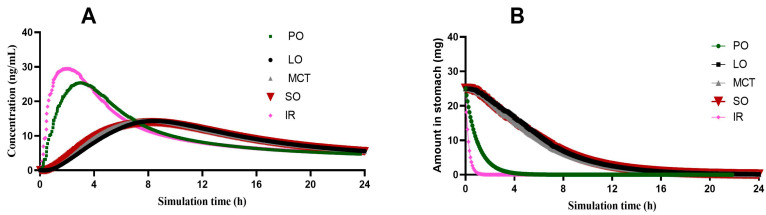
(**A**) The predicted concentration–time curves of prepared 1:4 CIN organogels in SO, LO and MCT. (**B**) Change in the amount of CIN in the stomach over time for 1:4 CIN organogels in SO, LO, MCT and PO, where IR represents the immediate-release tablet.

The constructed CIN model was then used to predict the pharmacokinetic parameters for the 1:4 CIN organogels. At this level of simulation, new inputs were essential, including the in vitro release and the floating time, as well as a change in the gastric residence time to 4 h for consideration of the floating time. Furthermore, the dosage form was proposed as a gastric-controlled system; except for the 1:4 CIN:PO organogel, which was simulated as an immediate-release dosage form due to its rapid release (within 1 h). As shown in Figure 8A, similar profiles were predicted as in vivo concentration–time curves for the 1:4 CIN organogels in SO, LO and MCT, while the 1:4 CIN:PO organogel presented a closer Cmax to the immediate-release 25 mg CIN tablet but a different AUC. This may be attributed to the slow release of CIN, which might initiate lysosomal entrapment leading to a decrease in the CIN plasma level. The pharmacokinetic parameters of all 1:4 CIN organogels in all oils are presented in Supplementary File S4.

In addition, Figure 8B presents the change in the amount of CIN in the stomach for all prepared organogels. Due to the rapid release of CIN from the 1:4 CIN:PO organogel, it exhibited a faster decrease in the CIN gastric content than the 1:4 CIN organogels in SO, LO and MCT, with a closer curve to that of the immediate-release CIN tablet.

Although successful in silico modeling was achieved using Gastroplus^®^, this study has a limitation regarding the validation of the results, considering which an in vivo study should be carried out to confirm the obtained pharmacokinetic data. Even though our model was executed on two populations of cinnarizine data and incorporated enzyme effects, in vivo research would allow for more accurate prediction of the inter-individual variability in population kinetics. Future works could address these limitations, along with further optimization of the formulation and methodology.

## 3. Conclusions

CIN was shown to be an excellent low-molecular-weight gelator, enabling successful gelation with PO, LO, MCT and SO. For all successful organogel formulas, experiments demonstrated their thermal reversibility, in situ gelation in water and considerable G′ values, indicating their suitability for oral administration. Moreover, the CIN organogels in all oils successfully floated in the release study, establishing their potential as a gastroretentive system. Furthermore, the potential of these dosage forms as a gastric-controlled system were validated through in silico simulations using the Gastroplus^®^ software with lysosomal entrapment, which helped to predict the associated pharmacokinetic parameters.

## 4. Materials and Methods

### 4.1. Materials

The CIN was kindly gifted from the Samraa Drug Industry, Iraq, while the PO, LO, MCT and SO were purchased from Bar-sur-Loup Pres Grasse (A.M) France, LOBA CHEMIE PVT. Ltd. (Colaba, Mumbai, India), Ketoandco (Kedah, Malaysia) and Xi’an Sonwu Biotech (Xi’an, Shaanxi, China), respectively.

### 4.2. Organogel Formulations

The organogels were prepared using CIN with the selected oils (PO, LO, MCT and SO), which are widely used as food additives and in pharmaceutical preparations [55]. A pilot study was carried out to determine a suitable ratio of CIN to oil. The initial ratio (1:10) was found to fail to result in organogel, as the gelator (CIN) concentration was too low. Hence, the proposed weight ratios of CIN:oil (mg) were 1:4, 1:6 and 1:8. The CIN and oils were heated in vials in a water bath at 90 °C for the different periods, following which the formulations were incubated at 90 °C for different periods (around 10 h for CIN:SO, 2 h for CIN:PO and LO, and 2 to 4 h for CIN:MCT). Then, the vials were allowed to cool at ambient temperature, and gelation was monitored before vial inversion. Upon vial inversion, if there was no flow, the CIN:oil mixture was classified as an organogel; meanwhile, if there was a flow, it was considered a liquid. The vial inversion experiment is the initial test to identify gelation through the absence of flow due to gravity force, providing evidence of a self-assembled network anchored to the bottom of the vial [56].

### 4.3. Thermoreversible Gelation and Organogel In Situ Formation Studies

The thermoreversibility of all successfully gelled organogel formulations was studied by incubating the organogel vials in a water bath at 90 °C for around 10 h for CIN:SO, 2 h for CIN:PO and LO, and 2 to 4 h for CIN:MCT. The formulations were then brought out from the water bath to monitor their gelation at ambient temperature (approximately 25 °C).

Additionally, the formation and ability to re-gel of the organogels was studied after they were liquefied by adding 50 microliters of ethanol to the 1:4 CIN organogels. These mixtures were then incubated in a water bath at 90 °C. Afterward, the organogels were poured into 200 mL of water at room temperature (approximately 25 °C) to observe their re-gelation process.

### 4.4. DSC

Small amounts of CIN organogels (ranging from 12 to 12.9 mg) were placed in a (T zero) pan with Hermetic lids. Using a DSC Mettler-Toledo (Columbus, OH, USA), the samples were heated from 0 to 300 °C at a rate of 10 °C/min. The STARe excellence thermal analysis software was used to evaluate the melting peaks.

### 4.5. Oscillatory Rheology Study

The Anton Paar MCR 302 rheometer (Graz, Austria) and a parallel plate (PP 25/SN 61895) were used to perform the amplitude and frequency sweep studies.

#### 4.5.1. Amplitude Sweep

The organogels were removed from the vials and placed between the plates using the PP 25 geometry. The operating parameters were set to apply an angular frequency of 10 rad/s, a temperature of 37 °C, and a strain range of 0% to 100%. The Rheoplus software (Version: 2.6x) was used to calculate the linear viscoelastic region (LVER) and flow point. This test enabled identification of the organogels’ viscoelasticity according to the parameters G′ (storage modulus), G″ (loss modulus), LVER (linear viscoelastic region) and flow point. The G′ value reflects the strength or the elasticity of the organogel, while the G″ value identifies the organogel’s liquid status. Furthermore, the LVER value determines the organogel’s resistance to the applied augmented strain. The last parameter is the flow point, determined as the point where the values of G′ and G″ are equal, representing damage to the 3-dimensional network of the organogel.

#### 4.5.2. Frequency Sweep

At a selected strain in the linear viscoelastic region—which was one of the parameters determined in the amplitude sweep test—a frequency sweep analysis was performed on the organogels using the same geometry as in the amplitude sweep test, over a range of angular frequencies (0–100 rad/s) at 37 °C.

### 4.6. Fourier-Transform Infrared (FTIR)

Organogels or drops of oils were mixed with KBr and then compressed into a disc. The FTIR spectra for these discs were then captured using a Shimadzu 1800 spectrometer (Shimadzu Corporation, Kyoto, Japan) with beam wavelengths ranging from 400 to 4000 cm^−1^.

### 4.7. Optical Light Microscopy

Heated selected organogels were dropped onto a slide on a hot plate at 90 °C and then covered with a cover slip. These slides were allowed to cool down to ensure fiber formation, followed by examination under a microscope using the micro software with the camera MC500 (Lanoptik Technologies Ltd., Guangzhou, China) to capture images of the organogel scaffolds at a magnification of 4×.

### 4.8. In Vitro Release Study

The CIN organogel release studies were undertaken in a USP apparatus II. The paddles were rotated at 100 rpm in 900 mL of 0.1 N HCl (pH 1.2). A melted form of the organogels containing equivalent to 25 mg of cinnarizine was loaded into a hard gelatin capsule (size 00). The semi-solid form (also equivalent to 25 mg of cinnarizine) was then loaded into a hard gelatin capsule (size 00), which remained semi-solid within the capsule body. Samples of 5 mL were withdrawn at 0, 15, 30, 60, 120, 180 and 240 min. At each time point, 5 mL of 0.1 N HCl (pH 1.2) was added. The samples were analyzed using a UV–Vis spectrophotometer (UV-1650PC, Shimadzu Corporation, Kyoto, Japan) at λmax = 254 nm (y = 0.0557x + 0.0117; R^2^ = 0.997).

### 4.9. Physiologically Based Simulation of CIN Pharmacokinetics

The GastroPlus^®^ software (version 9.8.2, Simulations Plus Inc., Research Triangle Park, NC, USA) was used to construct a physiologically based pharmacokinetics (PBPK) oral absorption model for a 25 mg CIN immediate-release tablet. This model was then used to predict the pharmacokinetic parameters of CIN organogels from their in vitro release data. The Advanced Compartmental Absorption and Transit (ACAT) is the foundation of the GastroPlus^®^ software, which models the functions of the human gastrointestinal segments (stomach, duodenum, jejuna 1 and 2, ilea 1–3, caecum and ascending colon), controlled through many mathematical equations that correlate to the physiology of the GIT, including the gastric and intestinal residence times, release, absorption and drug disposition. The in silico model for CIN was built using predicted data (i.e., from the ADMET Predictor software) or literature data, as detailed in Table 1. All the enzyme inputs within the model were defined under the PBPK and/or gut option, as 2B6 and 1A2 have not yet been included in the gut segments within the Gastroplus^®^ 9.8.2 software. Additional inputs included in vitro release data after model construction to forecast the associated in vivo parameters. The validation of the physiological model was based on data obtained through a clinical study (fasting, healthy male human, 29 years old) taking a 25 mg immediate-release CIN tablet [57], and data extraction was performed with the aid of the GetData Digitizer version 2.26.0.2 software. All tissues were set as perfusion-limited. Verification of the model was performed based on the fold error value (to be no greater than 2) between the observed and predicted values of Cmax, Tmax, AUC_0-inf_ and AUC_0-t_, using the equations below: (a) the observed values greater than predicted, as well as the opposite case (b) [58,59].

-
Fold error=Observed valuePredicted value

-
Fold error=Predicted valueObserved value

## Data Availability

The original contributions presented in this study are included in the article. Further inquiries can be directed to the corresponding authors.

## Supplementary Materials

The following supporting information can be downloaded from https://www.mdpi.com/article/10.3390/gels12010058/s1, Supplementary File S1: Table S1. The gelation times of different CIN ratios in different oils. Supplementary File S2: Table S2. The amplitude sweep parameters G′, G″, LVER and flow points. The values represent the average of 3 trials ± (SD) at 37 °C. The angular frequency was set at 10 rad/s and the strain ranged from 0% to 100%. Supplementary File S3: Figure S3A. Whole FTIR spectra for the 1:4, 1:6 and 1:8 prepared organogels and the CIN; Figure S3B. The region from 1000 to 1700 cm^−1^ for all 1:4, 1:6 and 1:8 CIN organogels in all oils (PO, LO, MCT and SO). Supplementary File S4: Figure S4A. Parameter sensitivity analysis, revealing the effect of unbound fraction in enterocytes on Tmax and Cmax, as shown in A and B; Figure S4B. Gastroplus Figure for Stugeron^®^—observed (the dotted line) versus predicted (the solid line) using the same inputs for enzymes, permeability and lysosomal entrapment; Table S4 A. The observed and calculated pharmacokinetic parameters of 25 mg Stugeron® oral tablet with application of lysosomal entrapment; Table S4 B. Pharmacokinetic parameters of CIN organogels in oils.

## References

[1] Bonnet J., Suissa G., Raynal M., Bouteiller L. (2014). Organogel formation rationalized by Hansen solubility parameters: Dos and don’ts. J. Soft Matter.

[2] Yan N., Xu Z., Diehn K.K., Raghavan S.R., Fang Y., Weiss R.G. (2013). How do liquid mixtures solubilize insoluble gelators? Self-assembly properties of pyrenyl-linker-glucono gelators in tetrahydrofuran–water mixtures. J. Am. Chem. Soc..

[3] Martin B., Garrait G., Beyssac E., Goudouneche D., Perez E., Franceschi S. (2020). Organogel Nanoparticles as a New Way to Improve Oral Bioavailability of Poorly Soluble Compounds. Pharm. Res..

[4] Mohamed M.B.M., Dahabiyeh L.A., Sahib M.N. (2022). Design and evaluation of molecular organogel based on folic acid as a potential green drug carrier for oral route. Drug Dev. Ind. Pharm..

[5] Sanches S.C.d.C., Ré M.I., Silva-Júnior J.O.C., Ribeiro-Costa R.M. (2023). Organogel of acai oil in cosmetics: Microstructure, stability, rheology and mechanical properties. Gels.

[6] Sahoo S., Kumar N., Bhattacharya C., Sagiri S., Jain K., Pal K., Ray S., Nayak B. (2011). Organogels: Properties and applications in drug delivery. Des. Monomers Polym..

[7] Singh V.K., Pal K., Pradhan D.K., Pramanik K. (2013). Castor oil and sorbitan monopalmitate based organogel as a probable matrix for controlled drug delivery. J. Appl. Polym. Sci..

[8] Murdan S. (2005). Organogels in drug delivery. Expert Opin. Drug Deliv..

[9] Singh V.K., Pramanik K., Ray S.S., Pal K. (2015). Development and characterization of sorbitan monostearate and sesame oil-based organogels for topical delivery of antimicrobials. AAPS PharmSciTech.

[10] Pernetti M., van Malssen K., Kalnin D., Flöter E. (2007). Structuring edible oil with lecithin and sorbitan tri-stearate. Food Hydrocoll..

[11] Abdallah D.J., Weiss R.G. (2000). n-Alkanes gel n-alkanes (and many other organic liquids). Langmuir.

[12] Terech P., Friol S. (2007). Rheometry of an androstanol steroid derivative paramagnetic organogel. Methodology for a comparison with a fatty acid organogel. Tetrahedron.

[13] Maji S.K., Malik S., Drew M.G., Nandi A.K., Banerjee A. (2003). A synthetic tripeptide as a novel organo-gelator: A structural investigation. Tetrahedron Lett..

[14] Hao C., Gao J., Wu Y., Wang X., Zhao R., Mei S., Yang J., Zhai X., Qiu H. (2018). Design of folic acid based supramolecular hybrid gel with improved mechanical properties in NMP/H2O for dye adsorption. React. Funct. Polym..

[15] Giomini M., Giuliani A.M., Giustini M., Trotta E. (1991). Anthracycline gels: Preparation and some physico-chemical properties. Biophys. Chem..

[16] Lozano V., Hernández R., Ardá A., Jiménez-Barbero J., Mijangos C., Pérez-Pérez M.-J. (2011). An asparagine/tryptophan organogel showing a selective response towards fluoride anions. J. Mater. Chem..

[17] Kishida T., Fujita N., Hirata O., Shinkai S. (2006). Axial coordination changes the morphology of porphyrin assemblies in an organogel system. Org. Biomol. Chem..

[18] Haress N.G. (2015). Cinnarizine: Comprehensive Profile. Profiles Drug Subst. Excip. Relat. Methodol..

[19] Chu M.K., Wang S.-J. (2020). Nomenclature for flunarizine, cinnarizine, and lomerizine. Cephalalgia.

[20] Dyhrfjeld-Johnsen J., Attali P. (2019). Management of peripheral vertigo with antihistamines: New options on the horizon. Br. J. Clin. Pharmacol..

[21] Xavier R.P., Mengarda A.C., Silva M.P., Roquini D.B., Salvadori M.C., Teixeira F.S., Pinto P.L., Morais T.R., Ferreira L.L., Andricopulo A.D. (2020). H1-antihistamines as antischistosomal drugs: In vitro and in vivo studies. Parasit Vectors.

[22] Nagarwal R.C., Ridhurkar D.N., Pandit J. (2010). In vitro release kinetics and bioavailability of gastroretentive cinnarizine hydrochloride tablet. AAPS PharmSciTech.

[23] Arza R.A.K., Gonugunta C.S.R., Veerareddy P.R. (2009). Formulation and evaluation of swellable and floating gastroretentive ciprofloxacin hydrochloride tablets. AAPS PharmSciTech.

[24] Raghuvanshi S., Pathak K. (2014). Recent advances in delivery systems and therapeutics of cinnarizine: A poorly water soluble drug with absorption window in stomach. J. Drug Deliv..

[25] Varshosaz J., Tabbakhian M., Zahrooni M. (2007). Development and characterization of floating microballoons for oral delivery of cinnarizine by a factorial design. J. Microencapsul..

[26] Nigusse B., Gebre-Mariam T., Belete A. (2021). Design, development and optimization of sustained release floating, bioadhesive and swellable matrix tablet of ranitidine hydrochloride. PLoS ONE.

[27] Praveen R., Verma P.R.P., Venkatesan J., Yoon D.-H., Kim S.-K., Singh S.K. (2017). In vitro and in vivo evaluation of gastro-retentive carvedilol loaded chitosan beads using Gastroplus™. Int. J. Biol. Macromol..

[28] Hamdi D.S., Mohamed M.B.M. (2022). Formulation of metoclopramide HCl gastroretentive film and in vitro-in silico prediction using Gastroplus^®^ PBPK software. SPJ.

[29] Rogers M.A., Wright A.J., Marangoni A.G. (2009). Nanostructuring fiber morphology and solvent inclusions in 12-hydroxystearic acid/canola oil organogels. Curr. Opin. Colloid Interface Sci..

[30] Mohamed M.A., Attia A.K. (2017). Thermal behavior and decomposition kinetics of cinnarizine under isothermal and non-isothermal conditions. J. Therm. Anal. Calorim..

[31] Yilmaztekin M., Lević S., Kalušević A., Cam M., Bugarski B., Rakić V., Pavlović V., Nedović V. (2019). Characterisation of peppermint (*Mentha piperita* L.) essential oil encapsulates. J. Microencapsul..

[32] Guenet J.-M. (2021). Physical aspects of organogelation: A point of view. Gels.

[33] Aziz Z.Y., Mohsin M.B., Jasim M.H. (2023). Formulation and Assessment of Delayed/Slow-Release Diclofenac Sodium Edible Organogel Utilizing Low Molecular Weight Organogelators. Iraqi J. Pharm. Sci..

[34] Mohamed M.B.M., Qaddoori Z.S., Hameed G.S. (2022). Study the effect of 12-hydroxyoctadecanoic acid concentration on preparation and characterization of floating organogels using cinnarizin as modeling drug. Iraqi J. Pharm. Sci..

[35] Cerqueira M.A., Fasolin L.H., Picone C.S., Pastrana L.M., Cunha R.L., Vicente A.A. (2017). Structural and mechanical properties of organogels: Role of oil and gelator molecular structure. Int. Food Res..

[36] Zeng C., Wan Z., Xia H., Zhao H., Guo S. (2020). Structure and Properties of Organogels Developed by Diosgenin in Canola Oil. Food Biophys..

[37] Yeo L.K., Olusanya T.O., Chaw C.S., Elkordy A.A. (2018). Brief effect of a small hydrophobic drug (cinnarizine) on the physicochemical characterisation of niosomes produced by thin-film hydration and microfluidic methods. Pharmaceutics.

[38] Martinez R.M., Oseliero Filho P.L., Gerbelli B.B., Magalhães W.V., Velasco M.V.R., da Silva Lannes S.C., de Oliveira C.L.P., Rosado C., Baby A.R. (2022). Influence of the Mixtures of Vegetable Oil and Vitamin E over the Microstructure and Rheology of Organogels. Gels.

[39] Patel A.R., Babaahmadi M., Lesaffer A., Dewettinck K. (2015). Rheological profiling of organogels prepared at critical gelling concentrations of natural waxes in a triacylglycerol solvent. J. Agric. Food Chem..

[40] Guenet J.-M. (2016). Organogels: Thermodynamics, Structure, Solvent Role, and Properties.

[41] Shakeel F., Kazi M., Alanazi F.K., Alam P. (2021). Solubility of cinnarizine in (Transcutol+ water) mixtures: Determination, Hansen solubility parameters, correlation, and thermodynamics. Molecules.

[42] Kirtane A.R., Karavasili C., Wahane A., Freitas D., Booz K., Le D.T.H., Hua T., Scala S., Lopes A., Hess K. (2022). Development of oil-based gels as versatile drug delivery systems for pediatric applications. Sci. Adv..

[43] Lupi F., Mancina V., Baldino N., Parisi O., Scrivano L., Gabriele D. (2018). Effect of the monostearate/monopalmitate ratio on the oral release of active agents from monoacylglycerol organogels. Food Funct..

[44] Shahba A.A.-W., Mohsin K., Alanazi F.K. (2012). Novel self-nanoemulsifying drug delivery systems (SNEDDS) for oral delivery of cinnarizine: Design, optimization, and in-vitro assessment. AAPS PharmSciTech.

[45] Halaby R. (2019). Influence of lysosomal sequestration on multidrug resistance in cancer cells. CDR.

[46] Llanos S., Megias D., Blanco-Aparicio C., Hernández-Encinas E., Rovira M., Pietrocola F., Serrano M. (2019). Lysosomal trapping of palbociclib and its functional implications. Oncogene.

[47] Dos Santos E.M., Ferraz H.G., Issa M.G., Duque M.D. (2023). Development of extended-release formulations containing cyclobenzaprine based on physiologically based biopharmaceutics modeling and bioequivalence safe space. J. Pharm. Sci..

[48] Sheue Nee Ling S., Magosso E., Abdul Karim Khan N., Hay Yuen K., Anne Barker S. (2006). Enhanced oral bioavailability and intestinal lymphatic transport of a hydrophilic drug using liposomes. Drug Dev. Ind. Pharm..

[49] Nowacka-Krukowska H., Rakowska M., Neubart K., Kobylińska M. (2007). High-performance liquid chromatographic assay for cinnarizine in human plasma. Acta Pol. Pharm..

[50] Abouelatta S.M., Aboelwafa A.A., Khalil R.M., El-Gazayerly O.N. (2016). Utilization of ionotropic gelation technique for bioavailability enhancement of cinnarizine: In-vitro optimization and in-vivo performance in human. Drug Deliv..

[51] Santos J., Lobato L., Vale N. (2021). Clinical pharmacokinetic study of latrepirdine via in silico sublingual administration. In Silico Pharmacol..

[52] Kirtane M.V., Bhandari A., Narang P., Santani R. (2019). Cinnarizine: A contemporary review. Indian J. Otolaryngol. Head Neck Surg..

[53] Kariya S., Isozaki S., Uchino K., Suzuki T., Narimatsu S. (1996). Oxidative metabolism of flunarizine and cinnarizine by microsomes from B-lymphoblastoid cell lines expressing human cytochrome P450 enzymes. Biol. Pharm. Bull..

[54] Chen Y., Li X., Wei X., Gou J., Tang X., He H., Xu H. (2017). Influence of lipid composition on the oral bioavailability of cinnarizine sub-microemulsions. Eur. J. Lipid Sci. Technol..

[55] Özyazici G. (2022). New Development on Medicinal and Aromatic Plants-II.

[56] Chivers P.R., Smith D.K. (2019). Shaping and structuring supramolecular gels. Nat. Rev. Mater..

[57] Christiansen M.L., Holm R., Abrahamsson B., Jacobsen J., Kristensen J., Andersen J.R., Müllertz A. (2016). Effect of food intake and co-administration of placebo self-nanoemulsifying drug delivery systems on the absorption of cinnarizine in healthy human volunteers. Eur. J. Pharm. Sci..

[58] Chen F., Liu H., Wang B., Yang L., Cai W., Jiao Z., Yang Z., Chen Y., Quan Y., Xiang X. (2020). Physiologically based pharmacokinetic modeling to understand the absorption of risperidone orodispersible film. Front. Pharmacol..

[59] Wang L., Chen J., Chen W., Ruan Z., Lou H., Yang D., Jiang B. (2023). In silico prediction of bioequivalence of atorvastatin tablets based on GastroPlus™ software. BMC Pharmacol. Toxicol..

