# Supplementary material for: Supramolecular Organogels Based on Cinnarizine as a Potential Gastroretentive System: In Vitro and In Silico Simulations
Source: Gels. 2026 Jan 8;12(1):58. doi: 10.3390/gels12010058 (PMC12841481; doi:10.3390/gels12010058)
Supplement: Supplementary file 1 [file gels-12-00058-s001.zip › supplementary File S4.pdf]

## supplementary File S4

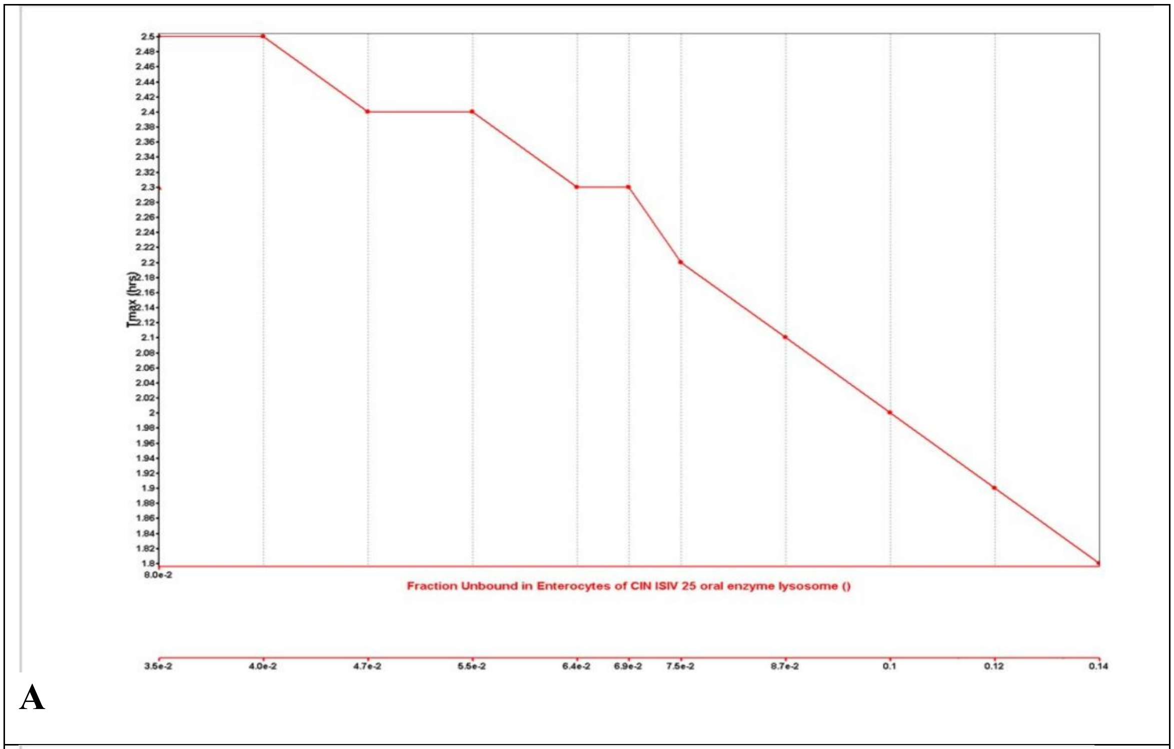


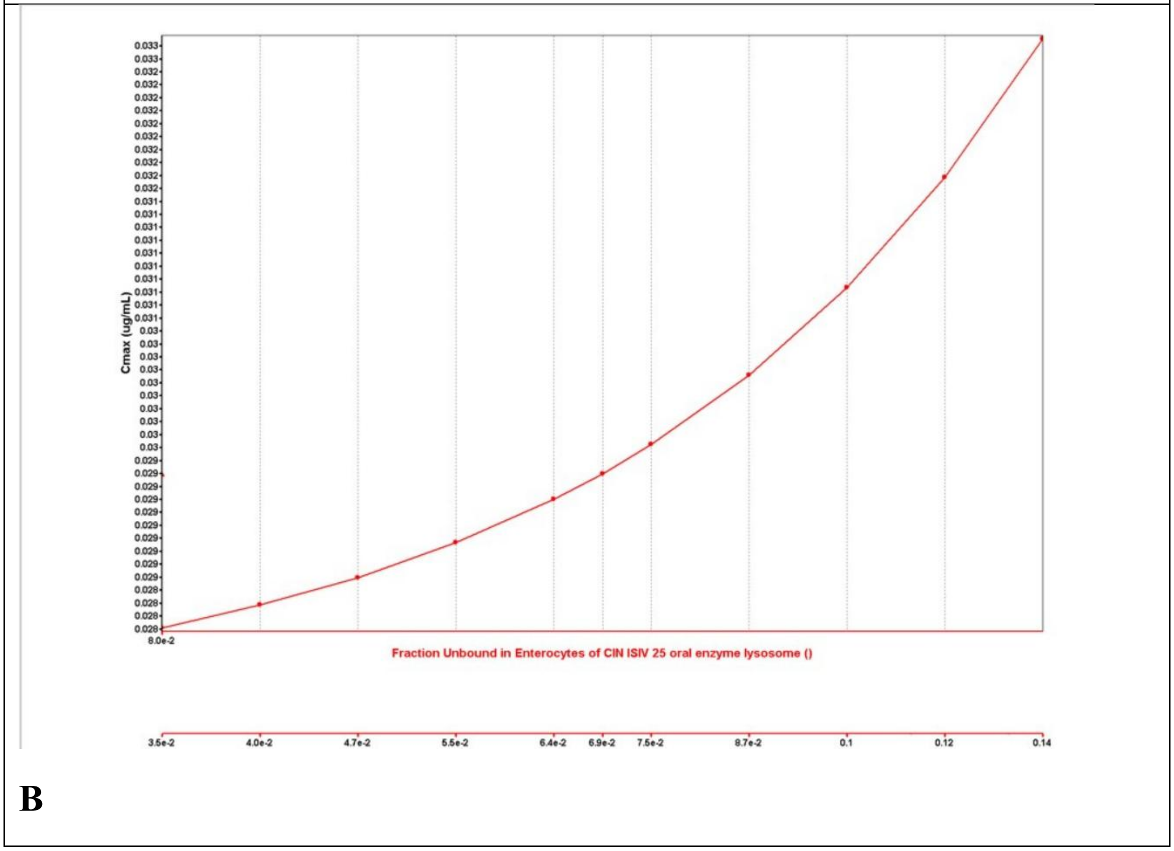


**Figure S4 A. Parameter sensitivity analysis revealing the effect of fraction unbound of enterocytes on Tmax and Cmax as shown in A and B.**

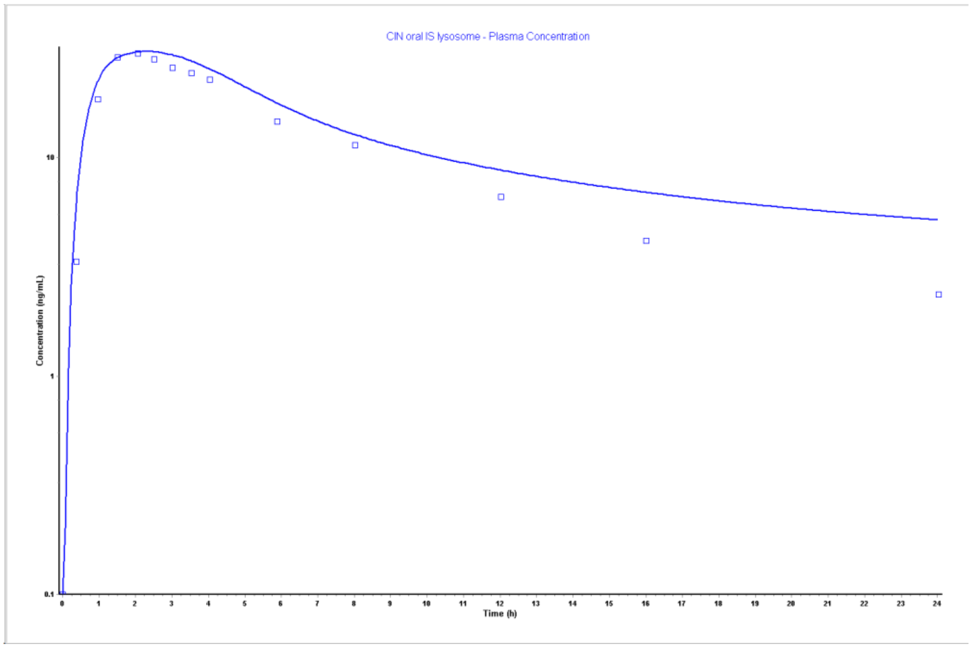


**Figure S4 B. Gastroplus Figure for Stugeron® observed the dotted line versus predicted the solid line using the same inputs of enzymes, permeability and lysosomal entrapment.**

**Table S4 A. The observed and calculated pharmacokinetics parameter of 25 mg Stugeron® oral tablet with application of lysosomal entrapment.**

| Pharmacokinetics parameters (units) | Observed Values Stugeron® | Calculated values | Fold error |
|---|---|---|---|
| Cmax (ng/ ml) | 29.921 | 30.624 | 1.02 |
| Tmax (h) | 2.065 | 2.32 | 1.12 |
| AUC $_{0\text{-inf}}$ | 267.5 | 481.15 | 1.79 |
| AUC $_{0\text{-t}}$ (ng-h/mL) | 233.85 | 289.21 | 1.23 |

**Table S4 B. Pharmacokinetics parameters of CIN organogels in oils.**

| Formulation of CIN | Cmax (ng/mL): | Tmax (h) | AUC $_{0\text{-inf}}$ (ng-h/mL): | AUC $_{0\text{-t}}$ (ng-h/mL): |
|---|---|---|---|---|
| 1:4 PO | 25 | 3.28 | 421.98 | 258.26 |
| 1:4 SO | 13.914 | 8.24 | 335.24 | 215.13 |
| 1:4 MCT | 14.44 | 7..84 | 336.88 | 216.64 |
| 1:4 LO | 14.328 | 8.4 | 332.74 | 213.32 |
