# Supplementary material for: Supramolecular Organogels Based on Cinnarizine as a Potential Gastroretentive System: In Vitro and In Silico Simulations
Source: Gels. 2026 Jan 8;12(1):58. doi: 10.3390/gels12010058 (PMC12841481; doi:10.3390/gels12010058)
Supplement: Supplementary file 1 [file gels-12-00058-s001.zip › supplementary File S1.pdf]

supplementary File S1

Table S1. The gelation times of different CIN ratios in different oils

Oil	MCT			SO			PO			LO		
Organogel ratios	1:4	1:6	1:8	1:4	1:6	1:8	1:4	1:6	1:8	1:4	1:6	1:8
Gelation time in h.	1	3	6	3	12	12	4	8	10	48	144	--
