# Supplementary material for: Supramolecular Organogels Based on Cinnarizine as a Potential Gastroretentive System: In Vitro and In Silico Simulations
Source: Gels. 2026 Jan 8;12(1):58. doi: 10.3390/gels12010058 (PMC12841481; doi:10.3390/gels12010058)
Supplement: Supplementary file 1 [file gels-12-00058-s001.zip › supplementary File S2.pdf]

Supplementary File S2

Table S2. The amplitude sweeps parameters G', G'', LVER and flow points, as the values represent the average of 3 trials ± (SD) at 37 °C, the angular frequency set at 10 rad/s and the strain range of 0% to 100%

Formula name	G'± (SD) (Pa)	G''± (SD) (Pa)	LVER ± (SD) (%)	Flow point ± (SD) (%)
1:4 PO	249906	42613	0.023067	3 (1.732)
	(53350)	(2717)	(0.009324)	
1:6 PO	227263	30584	0.0273	1.466
	(97597)	(15181)	0.0024	(0.0577)
1:8 PO	146190	23725	0.02555	0.9
	(3162)	(3703)	0.0236	0.173205
1:4 SO	1321600	173523	0.0326	2.166
	(248501)	(82704)	(0.0038)	(0.288)
1:6 SO	769470	69557	0.0296	1.366
	(455984)	(31032)	(0.0014)	(0.115)
1:8 SO	432050	45418	0.027	1.633
	(208627)	(25534)	(0.0004)	(0.321)
1:4 MCT	1410266	271410	0.026	5.833
	(239514)	(108255)	(0.0088)	(4.3108)
1:6 MCT	806420	73857	0.029	2.333
	(83884)	(7940)	(0.0008)	(0.577)
1:8 MCT	172156	19577	0.027	1.433
	(15998)	(5990)	(0.0001)	(0.0577)
1:4 LO	1016113	113463	0.028	1.5
	(109074)	(17047)	(0.0014)	(0.5)
1:6 LO	157093	20265	0.027	2.333
	(31671)	3668	(0.0008)	(1.154)
