# Supplementary material for: Supramolecular Organogels Based on Cinnarizine as a Potential Gastroretentive System: In Vitro and In Silico Simulations
Source: Gels. 2026 Jan 8;12(1):58. doi: 10.3390/gels12010058 (PMC12841481; doi:10.3390/gels12010058)
Supplement: Supplementary file 1 [file gels-12-00058-s001.zip › supplementary File S3.pdf]

supplementary File S3

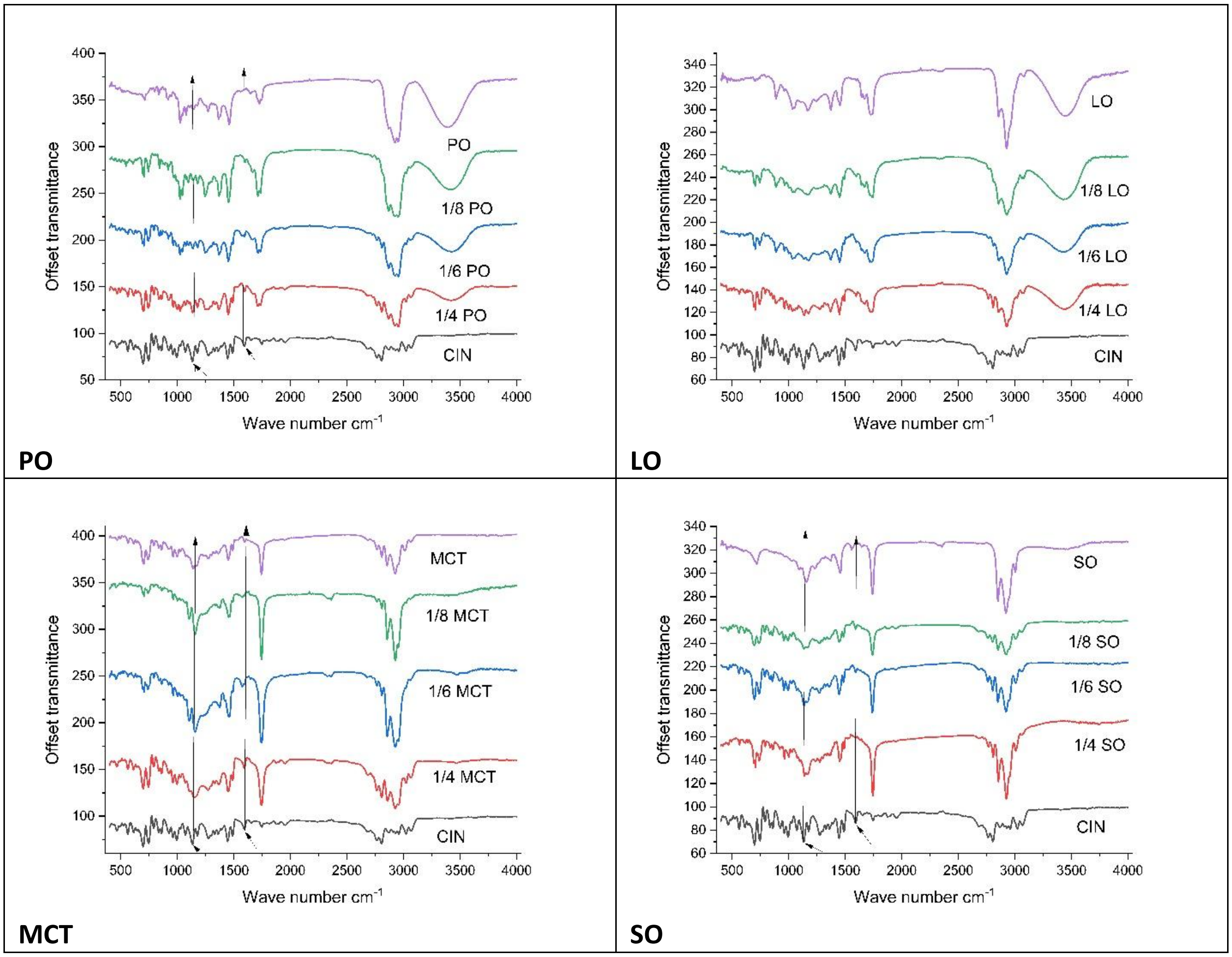

Figre S3 A. The whole FTIR spectra for the 1:4, 1:6 and 1:8 prepared organogels and the CIN

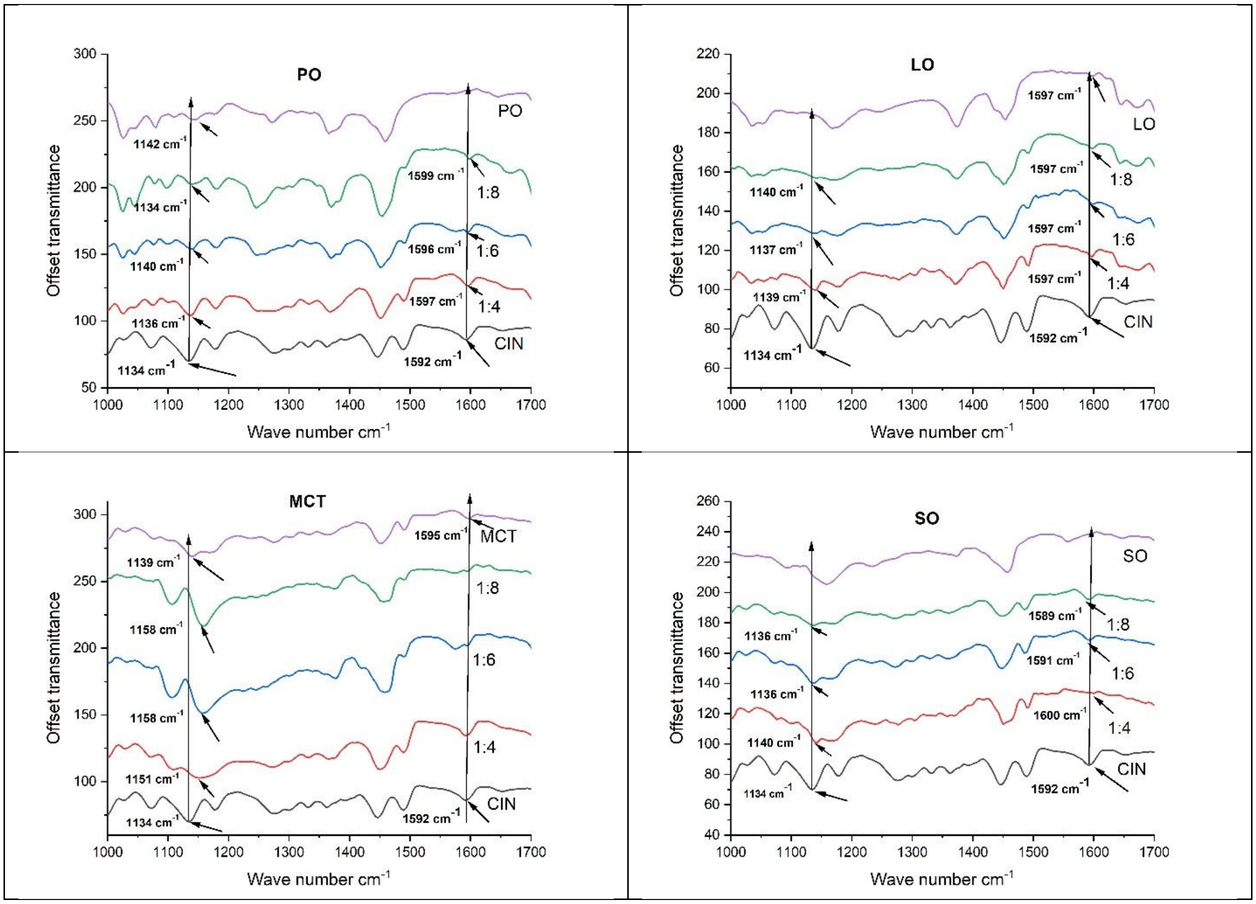

Figure S3 B. The region from 1000 cm^{-1} and 1700 cm^{-1} for all 1:4, 1:6 and 1:8 CIN organogels in all oils PO, LO, MCT and SO.
